# Videolaryngostroboscopic analysis of patients submitted to radiation therapy for the treatment of glottic cancer

**DOI:** 10.1590/S1808-86942010000100009

**Published:** 2015-10-17

**Authors:** André Luís Quarteiro, Rogério Aparecido Dedivitis, Elio Gilberto Pfuetzenreiter

**Affiliations:** 1 Otorhinolaryngology specialist, master's degree student, graduate course on health science, Heliopolis Hospital (HOSPHEL), Sao Paulo; 2 Physician, associate professor, Lusiada Foundation (UNILUS); 3 Physician, master's degree student, graduate course on health science, HOSPHEL, Sao Paulo. Graduate course on health science, HOSPHEL, Sao Paulo

**Keywords:** laryngoscopy, laryngeal neoplasms, radiotherapy, voice

## Abstract

Radiation therapy is an adequate treatment for early laryngeal cancer, and it is important to study the vocal fold vibratory pattern, which is essential for a favorable voice outcome.

**Aim:**

To analyze laryngostroboscopic findings in a group of patients who underwent radiation therapy for the treatment of early glottic cancer.

**Methods:**

A retrospective study was conducted in order to evaluate 20 patients staged as T1a glottic tumors in the period from 1995 to 2005. A laryngostroboscopic protocol was applied.

**Results:**

Glottic closure was complete in 17 patients. The amplitude was normal in 14 treated vocal folds and in 18 contralateral vocal folds. The mucosa wave vibratory pattern was totally present in all vocal folds. The periodicity was always regular in all cases. The vestibular fold and arytenoid symmetry and movements were normal. There was occasional supraglottic lateral constriction in 4 cases. The mucosal appearance was normal in 14 and edematous in 6 patients. Mucus build up was observed in 12 patients.

**Conclusions:**

The vibratory pattern was normal or slightly diminished in both vocal folds after radiation therapy for early glottic cancer.

## INTRODUCTION

Stroboscopy is a sophisticated technique to evaluate, quantify and diagnose phonatory conditions; it was developed and is applied regularly and widely in many parts of the world. Social and scientific interest in human communication has been accompanied by significant developments in the diagnosis and treatment of laryngeal conditions.^1^

Treatment options for early glottis carcinoma include endoscopic removal of tumors, radiotherapy, and open laryngectomy techniques. Each option, including partial vertical laryngectomy, has its indication.^2^ Early glottic cancer may be defined as a carcinoma originating from the upper area of the glottis, not fixed to vocal folds. Tumors staged as T1 and T2 fall within this group. Controversies remain about treatment approaches and results, which may vary among institutions.^3^

Stroboscopy should be carried out ideally before and after surgery in those cases requiring surgical interventions involving the vocal folds.^4^ Radiotherapy may cause edema, atrophy, scars, supraglottic activity, and irregular free vocal fold borders. Stroboscopy generally reveals irregular closure and decreased or absent vibratory amplitude and mucosal waves.^5^

Radiotherapy as an oncologically appropriate option to treat early laryngeal glottic area cancer (T1a) requires an investigation of the vibratory pattern in these patients, since such vibrations are critical for the resulting voice. In this context, videolaryngostroboscopy is effective for this analysis.

The purposes of this study were to characterize the vibratory pattern in patients undergoing radiotherapy for the treatment of early glottic tumors by using videolaryngostroboscopy and to study the lesions encountered with this exam.

## METHOD

Twenty stroboscopic exam DVDs of patients undergoing radiotherapy only from January 1995 to December 2005 were evaluated. Twenty patients with a history of glottic epidermoid carcinoma, documented by histopathology of the primary tumor, all clinical stage T1a, undergoing radiotherapy only, were studied retrospectively. All patients had at least a 24-month post-radiotherapy interval. Patients with local recurrence of the disease or other laryngeal diseases were excluded. The Institutional Review Board in the city where the study was undertaken approved this study (number 007/2007).

The sample consisted of 18 males and 2 females, all Caucasian. Age ranged from 53 to 75 years (mean - 63 years). The duration of the clinical history ranged from three to 12 months; dysphonia was present in all patients. Clinical staging was T1a N0 M0. The radiotherapy dose ranged from 5000 to 7020 cGy (mean - 6300 cGy). No patient required nasogastric tube feeding or tracheostomy. No patient underwent speech therapy after treatment.

Videolaryngostroboscopy was done with topical anesthesia (10% lidocaine aspersion - Xilocaína®). A Karl Storz® 70o rigid telescope and a Kay Elemetrics® model RLS 9100 B stroboscope were used. The exam was done with comfortable and sustained voice emission (modal tone) of the vowel /e/. Two experts in stroboscopy analyzed the DVD data by consensus. Triple light emission frequency is: low velocity, high velocity, and locked, when voice and device frequency are identical, giving the impression of lack of vocal fold movement and vibration.

The following points were evaluated:

A) Aspect of the free border: the free border of the intermembranaceous portion of vocal folds is evaluated for straightness and smoothness, which should be present in vibration and breathing.
B) Glottic coaptation: may be complete or incomplete, and is determined by how much the vocal folds approximate during the close phase of voice emission in habitual frequencies and volumes. The evaluation is done during maximum vocal fold closure, when they reach the medial position.
C) Predominant glottic cycle phase: the open phase was considered as predominant when it took up more than two thirds of the glottic cycle. Similarly, the close phase was considered predominant when it took up more than two thirds of the glottic cycle.
D) Vertical approximation level: in the closed phase vocal folds should meet in the same vertical level.
E) Amplitude of movement: it is appropriate when it takes up half to two thirds of the transverse dimension of the vocal fold. The duration of the opening and closure phases, and the closed state should be evaluated and measured.
F) Mucosal wave: it is linear and uninterrupted, occurring parallel to the free border of vocal folds. It should be seen on the ventricular face of the vocal fold, and may absent or increased. It is absent when not seen on the vocal fold surface. It may be absent along the margin or in a restricted segment of the fold. It is decreased when present, but hard to observe, and restricted to the medial area of the vocal fold. It is normal when clearly seen moving from the longitudinal medial to lateral portion of the vocal fold. It is increased when moving from the contact point between vocal folds towards the lateral portion, moving over 50% of the vocal fold width.
G) Phase symmetry: the degree by which vocal folds are a mirror image of each other during vibration. Opening, closure and lateral extension of folds symmetry should be analyzed. Vocal folds are said to vibrate in phase, which is the normal condition, or to vibrate out of phase.
H) Periodicity: voice is quasi-periodic in sustained vowel emission. Periodicity is defined stroboscopically as the apparent regularity of ensuing vibratory cycles, as determined by stroboscopic light flash synchronicity. It is classified as: regular - the stroboscopic image is static; irregular - when successive cycles appear irregularly; and inconsistent - the vibratory pattern is mixed, with regular and irregular components.
I) Supraglottic structures: the aspect and mobility of supraglottic structures are observed. In normal conditions, these structures do not move significantly. They move significantly during compensatory hyperfunctional phonatory maneuvers. In such conditions, vocal fold approximation tends to be vestibular (medial constriction) and antero-posterior. In extreme cases, vestibular folds close in fully or the epiglottis may touch the arytenoids.

Our stroboscopic assessment protocol was based on the work of Hirano and Bless^6^ to which are added some parameters introduced by Weinstein et al.^7^ applied in postoperative assessments of supracrichoid partial laryn-gectomies. Additionally, the following vocal fold lesions were investigated: telangectasia,^8^ polypoid lesions, arytenoids edema, hyperemia, decreased vocal fold mobility^9^ and atrophy and scarring.^5^

## RESULTS

Frame 1 summarizes the results of videolaryngostroboscopy. The vocal fold borders affected by tumors and the contralateral vocal folds were smooth and straight in all 20 cases. Glottic coaptation was complete in 17 patients and incomplete in three; there was one case of a posterior triangular vocal gap in a female patient and anteroposterior fusiform gaps in two cases. The open phase predominated in four cases; there was no predominant phase in eight exams; the closed phase predominated in eight other cases. The vertical approximation level was equal for both vocal folds in all cases. In the affected vocal folds we found that the amplitude was normal in 14 cases, slightly diminished in four cases, and moderately diminished in two cases. In the contralateral vocal fold, the amplitude was normal in 18 cases; it was diminished discretely in two cases. The mucosal wave was normal in 18 cases in affected vocal folds; it was nearly absent in one case, and moderately absent in one case. It was normal in the contralateral vocal fold in 19 cases, and nearly absent in one case. Vibration occurred fully in both vocal folds. Phase symmetry was regular in 17 cases; it was generally regular in two patients, and always irregular in one. Periodicity was regular in all cases. Movement symmetry of vestibular folds was equal in all cases. Movement was normal in all cases. Movement symmetry of the arytenoids was equal in all assessments. Movement was normal in all cases. Hyperfunction was absent in 16 evaluations, and occasionally present in four cases. The site of the mucosal wave was the glottis in 18 cases, and mixed in two evaluations. The mucosa was normal in 14 cases, and edematous in six evaluations. The shape of the epiglottis was straight in 13 evaluations, and crescent-shaped in 7 cases. No mucus was seen in eight cases; balls of mucus were seen in 12 evaluations.

**Frame 1:** Results of videolaryngostroboscopy.

Border of vocal fold						
	smooth/straight	2	3	4	rough/irregular	
Compromised	20	0	0	0	0	
Contralateral	20	0	0	0	0	
Glottic coaptation						
Complete	anteroposterior fusiform gap	posterior triangular gap				
17	2	1				
Phase closure						
normal	open	closed				
8	4	8				
Vertical level						
equal	compromised lesser	contralateral lesser	questionable	impractical		
20	0	0	0	0		
Amplitude						
	normal	+	++	+++	no movement	impractical
compromised	14	4	2	0	0	0
contralateral	18	2	0	0	0	0
Mucosal wave						
	normal	+	++	+++	absent	impractical
compromised	18	1	1	0	0	0
contralateral	19	1	0	0	0	0
Vibratory behavior						
	full presence always	full presence occasional	partial absence always	full absence occasional	full absence always	impractical
compromised	20	0	0	0	0	0
contralateral	20	0	0	0	0	0
Phase symmetry						
regular	generally regular	generally irregular	always irregular	impractical		
17	2	0	1	0		
Periodicity						
regular	generally regular	generally irregular	always irregular			
20	0	0	0			
Vestibular folds - movement symmetry						
equal	compromised > contralateral	contralateral > compromised				
20	0	0				
Vestibular folds - movement						
normal	compromised+	compromised++	fully compromised			
20	0	0	0			
Arytenoid - movement symmetry						
igual	compromised > contralateral	contralateral > compromised				
20	0	0				
Arytenoids - movement						
normal	good	poor				
20	0	0				
Hyperfunction						
absent	occasionally present	present				
16	4	0				
Site of mucusal wave						
glottis	supraglottis	mixed				
18	0	2				
Aspect of mucosa						
normal	edematous	humid	dry			
14	6	0	0			
Shape of epiglottis						
straight	crescent-shaped	omega shaped				
13	7	0				
Mucus formation						
not observed	observed anterior	posterior	mucus ball	marginal		
8	0	0	12	0		

Table 1 shows other lesions that were found in the endoscopic exams: telangiectasia in 16 cases; hyperemia in 12; vocal fold “8” atrophy; polypoid lesions in fives; and arytenoid edema in two cases.

**Table 1:** Lesions found on laryngostroboscopy.

Lesion	n	%
telangectasia	16	80
polypoid lesion	5	25
arytenoid edema	2	10
hyperemia	12	60
decreased vocal fold mobility	0	0
atrophy	8	40
scar	0	0

## DISCUSSION

Radiotherapy is generally preferred as the first treatment for early glottic carcinoma. This is based mostly on its superior results of voice quality, which is maintained in patients undergoing radiotherapy compared to those treated primarily with surgical preservation of the larynx, as well as its high cure rates. Minor complications of irradiation range from oropharyngeal mucositis to mild laryngeal edema.^10^

Changes of voice are due to tissue damage. Visual morphological changes during radiotherapy may be described as: (1) increased vocal fold size and tumor mass before radiotherapy; (2) increased size and mass of tumor immediately after radiotherapy due to the resulting edema; and (3) decreased edema after some time has elapsed, such as decreased secondary reactions.^11^ The acute effects of radiotherapy have also been described as: erythema; mucosal desquamation; mucositis; mild vocal fold and arytenoids edema; temporary coughing and persistent hoarseness. In the long term (one to seven years after radiotherapy), there may also be tissue necrosis; condronecrosis; poorly functioning salivary glands; severe vocal fold, arytenoids and vestibular fold edema; dysphagia; and severe dysphonia. We found: telangectasia in 16 cases (80%); hyperemia in 12 cases (60%); vocal fold atrophy in eight cases (40%); polypoid lesions in five cases (25%); arytenoids edema in two cases (10%). The irradiation dose of our patients ranged from 5000 to 7020 cGy (mean - 6300 cGy), which was a high dose.

Although radiotherapy results show a high rate of local control and preservation of voice in early stage glottic carcinoma, the ideal radiotherapy dose plan is still debated. Long term effects included, in 90 T1 and T1 patients, moderate dysphonia in 14 patients (16%) and frequent moderate to severe laryngeal edema in nine patients (10%). No patient developed ulcers or cartilage necrosis. It is possible that the size of the fraction that was used may have increased the susceptibility to surgical complications.^12^ Head and neck carcinomas are often treated with radiotherapy (60–68 Gy in 30–34 fractions), and early and late complications are inevitable. Acute reactions develop during and immediately after therapy, which may change voice. Such early collateral effects are reversible and may be relieved in a variety of ways. Late collateral effects develop months to years after therapy, and are generally irreversible. The severity of collateral effects depends on the total radiation dose, its fractioning and irradiated volume.^13^

Reported complications of radiotherapy are: early complications (mucositis and skin desquamation) and late complications (fibrosis, laryngeal necrosis, chronic edema with insufficient passage of air - inadequate voice and sto-mal stenosis).^14^ Patients that continue to smoke appear to develop late edema, possibly as chronic laryngitis in a context of deficient lymphatic drainage.^9^,^14^ Another common late complication of irradiation is telangectasia on vocal folds; this was not a clinical problem in any of the study cases. Mild dysphonia was also seen in some patients, possibly due to vocal fold changes due to the tumor itself, biopsy trauma, and mild post-irradiation changes.^8^ Factors that have been identified as degrading voice quality after irradiation are smoking in large quantities, radiotherapy, and ample biopsies.^15^

Glottic tumors result in asymmetrical and decreased elasticity of the affected vocal fold, leading to a delay in vibration with non-synchronized movement. Voice improves after the tumor is removed. After radiotherapy, the tumor mass is replaced by tissue that is less flexible than healthy tissues. Videolaryngostroboscopy showed vibration loss involving both vocal folds in some patients, probably due to submucosal fibrosis due to irradiation affecting the underlying stroma.^16^ In this study, the full vibratory behavior was found in both vocal folds - with disease and the contralateral healthy fold - in all cases. However, the amplitude and the mucosal wave were stronger in the contralateral vocal fold. In affected vocal folds, the amplitude was normal in 14 cases, slightly diminished in four cases, and moderately diminished in two cases. In the contralateral fold, the amplitude was normal in 18 evaluations, and mildly diminished in two cases. Similarly, in the affected vocal folds, the mucosal wave was normal in 18 cases, slightly absent in one case, and moderately absent in one other case. In the contralateral vocal fold, it was normal in 19 evaluations, and slightly absent in one case. Radiotherapy destroys tumors causing minimal scarring; but radiation also affects the contralateral vocal fold. Thus, videolaryngostroboscopy generally shows a glottic wave tilted to the contralateral side. The effect of radiation on the healthy vocal fold may be strong enough to counter-balance the less traumatic effect of radiation on tumors.

In 26 patients undergoing radiotherapy (50 Gy, in 2.5 Gy fractions, for early T1-T2 glottic carcinoma), nine had local complications (35%), five had vocal fold erythema or edema, and three developed vocal fold scars.^17^ Scarring occurred in 18 radiotherapy-treated patients with Tis-T1 vocal fold carcinomas, which could result in fibrosis and increase vocal fold tension. Other studies, however, have shown that 80% of these patients recovered their voice within four months of irradiation, 12% recovered their voice after six or more months of irradiation, and the remaining 8% truly develop voice with their voice.^18^

Of 78 patients that achieved local control of stage I glottic carcinoma after radiotherapy, 11 (14%) had minor chronic complications; severe complications requiring surgery were not encountered. Minor chronic complications included laryngitis in one patient, arytenoids edema lasting over six months following radiotherapy in one patient, decreased vocal fold mobility in four patients, and polypoid lesions without recurrences in seven patients.^9^

Complications of more significant impact after radiotherapy are long-term arytenoids edema (at times requiring tracheostomy) and cartilage necrosis. These complications vary according to the type of daily radiation fraction. A 1 to 2% incidence of severe complications is acceptable in early phases of glottic tumors.^19^ Tracheostomy was not required in any of our cases, and there was no clinically significant cartilage necrosis.

Videolaringostroboscopy shows rigidity and scarring on the affected side; in several patients with no previous surgery, the contralateral vocal fold had a normal mucosal wave. Radiation resulted in diffuse bilateral rigidity. Voice after radiotherapy was considered abnormal, reflecting diffuse glottic rigidity.^20^ Radiomucositis results in diminished vibration amplitude in both vocal folds.^21^ Vibration and the mucosal wave recovered gradually, and glottic coaptation once again became complete, from one to three months after the end of radiotherapy. Stroboscopy showed that all patients had a mucosal wave six months after radiotherapy.^22^ Laryngostroboscopy showed excellent movement and minor vocal fold irregularities, with a significantly improved glottic wave, in the irradiated group compared to the operated group (p<0.005).^16^

Vibration amplitude is defined as the extent of horizontal excursion of vocal folds in their movement. Each fold should be assessed independently and then compared. Amplitude is decreased when the mucosa is altered due to the mass, tension, and rigidity. Freezing a recorded image makes it possible to measure vibration amplitude. The relation between the open and closed phase of the glottic cycle has functional implications. The open phase takes up from 50 to 70% of the glottic cycle, depending on phonatory frequency and intensity. The open phase may predominate if the mucosa is rigid, and the closed phase may predominate in hyperfunctional disorders. Vocal folds have an elliptical shape in maximal lateral excursion; in their normal state, however, vocal folds should be straight and free or irregularities. Irregularities along their borders range from mild to extreme.^3^

A laryngeal videostroboscopic study for radiotherapy revealed that although there were cases in which the vibration amplitude of mucosal waves suggested more restriction on one side, there were no cases in which vibration amplitude was decreased in one vocal fold and normal in the other. Glottal gap, decreased vocal fold mass, and supraglottic hyperfunction results were significantly correlated. Common sequelae include decreased vocal fold microcirculation, loss of small salivary glands in the larynx, mucositis, odynophonia, xerostomy, mild fibrosis, edema, and telangiectasia, all of which cause dysphonia. Incomplete glottic coaptation may result in turbulent flow, which may essentially add to dysphonia, insufficient volume, and perception of voice effort. Common results of increased vascularity and decreased amplitude of mucosal wave vibration suggest a rigid vocal fold mucosa, which also may result in a glottal gap.^23^

Like any method, stroboscopy is also limited. In our context, the first limitation is the high cost of this device for most laryngology specialists. The exam itself has the same limitations as conventional laryngoscopy. The most frequent causes of an incomplete exam are: patients may find it impossible to sustain a constant and firm tone of voice to trigger stroboscopic light; decreased phonation time, which limits the minimal required time for analyzing stroboscopic parameters; the presence of secretions; patients with excessive reflexes; excessive supraglottic activity; lack of cooperation from patients; impossibility of eliminating the artistic vibrato; and the presence of large legions causing a mass effect, such as polyps, which may not allow an adequate view of the glottic area. The vibratory pattern may be affected if patients do not follow the examiner's instructions, if they are anxious about the exam, fatigued or with other health issues. Anxiety may simulate a hyperfunctional disorder, about which clinicians should be aware, repeating the exam if necessary. The telescope may increase tension, since the exam is done while pulling the tongue, a non physiological state.3 Stroboscopy reveals the behavior of vocal folds in vibration, but interpretation remains subjective and depends on the experience of examiners.

## CONCLUSION

Vibration patterns were normal or mildly decreased in both vocal folds after radiotherapy for T1a glottic cancer. We found: telangectasia in 16 cases; hyperemia in 12 cases; vocal fold atrophy in eight cases; polypoid lesions in five cases; and arytenoids edema in two cases.
